# Frontal Delta Dissimilarity During Moral Persuasion: Insight from an EEG Hyperscanning Study

**DOI:** 10.3390/brainsci15121302

**Published:** 2025-12-02

**Authors:** Roberta A. Allegretta, Angelica Daffinà, Michela Balconi

**Affiliations:** 1 International Research Center for Cognitive Applied Neuroscience (IrcCAN), Università Cattolica del Sacro Cuore, 20123 Milan, Italy; robertaantonia.allegretta1@unicatt.it (R.A.A.); michela.balconi@unicatt.it (M.B.); 2 Research Unit in Affective and Social Neuroscience, Department of Psychology, Università Cattolica del Sacro Cuore, 20123 Milan, Italy

**Keywords:** moral decision-making, persuasive interaction, EEG, delta, hyperscanning

## Abstract

**Background/Objectives**: Persuasive communication in moral decision-making contexts involves complex emotional and cognitive processes. This study aimed to investigate electrophysiological (EEG) dissimilarity between individuals during a persuasive interaction on a moral dilemma. **Methods**: Participants were paired into 14 dyads in which a member assumed the role of Persuasive Agent (PA) and the other of Persuasion Target (PT), discussing a moral decision-making scenario while their neural activity was recorded through an EEG hyperscanning paradigm. Dyads were later categorized based on perceived viewpoint change (high, mixed, low), and dissimilarity within dyads in EEG bands was analyzed across frontal, temporo-central, and parieto-occipital regions in left and right hemispheres. **Results**: Results showed a significant increase in frontal delta-band dissimilarity in mixed dyads, compared to temporo-central and parieto-occipital areas. The greater frontal delta dissimilarity in mixed dyads likely reflects divergent emotional and motivational engagement during persuasion. Specifically, individuals who changed their viewpoint may have exhibited stronger emotional resonance and attentional engagement compared to their partner. **Conclusions**: The study advances understanding of the neural mechanisms underlying persuasion in morally charged contexts and offers new insights into dyadic brain dynamics during complex social exchanges.

## 1. Introduction

In a world increasingly shaped by moral debates—on climate change, human rights, public health, etc.—the ability to persuade others of what is “right” or “wrong” holds significant social and political power. Moral persuasion is not just about presenting facts; it often involves appealing to values, emotions, and shared norms [1,2]. These persuasive dynamics become especially relevant in everyday situations where individuals, particularly those in positions of authority (such as politicians or managers), are required to make decisions that can deeply affect other people’s lives.

Some of these decisions can be classified as moral, as they involve evaluating what is fair, just, or ethically appropriate. Indeed, such scenarios demand a multifaceted process of reflection, including assessment, judgment, and determination of whether an action aligns with ethical standards [3,4]. Moral decision-making often takes the shape of a moral dilemma—a short narrative that describes a situation marked by a conflict between opposite moral principles [5,6]. Such a conflict occurs when a person faces two opposing courses of action, each supported by its own ethical reasoning, and must confront the fact that choosing one will exclude the other. In these situations, both options typically carry strong and legitimate moral justifications, making the decision particularly complex and emotional [6].

Understanding how persuasion functions within these moral contexts is crucial for analyzing how opinions are formed, how they shift, and how moral decisions are justified or challenged in both public and private spheres. To this purpose, neuroscience plays a crucial role in deepening our understanding of the persuasive processes underlying moral decision-making and reasoning, as it allows researchers to investigate internal mental mechanisms that are not directly observable. By employing advanced techniques to monitor neural activity in real time, neuroscience provides valuable insights into cognitive functions that often operate below the level of conscious awareness and are difficult to express verbally [7,8,9]. Moreover, to better grasp the complexity of interpersonal dynamics during real-time social interactions, neuroscience has increasingly adopted the “hyperscanning” paradigm, marking a shift from traditional single-subject designs to what is now referred to as “two-person neuroscience.” This approach enables the simultaneous recording of neural activity from two or more individuals as they engage in interaction, offering valuable insights into the shared and reciprocal nature of social cognition [10,11,12,13].

Studies focusing on the neural correlates of persuasion [14] have revealed that central to this process is the medial prefrontal cortex (mPFC), particularly the ventromedial prefrontal cortex (vmPFC), which integrates emotional and cognitive evaluations and predicts behavioral change, especially in response to personalized messages. The amygdala contributes to processing the emotional salience of persuasive content, while the temporoparietal junction (TPJ) and medial temporal areas are involved in understanding others’ perspectives [15]. The dorsolateral prefrontal cortex (DLPFC) supports cognitive control and rational evaluation, and the precuneus and posterior cingulate cortex are linked to self-referential thinking. Together, these regions form the neural foundation of how persuasive messages are processed, evaluated, and potentially lead to attitude or behavior change. More interestingly, studies examining neural coupling between the persuader and the receiver revealed that persuasive arguments significantly enhanced neural coupling in frontal areas [e.g., superior frontal gyrus (SFG) and inferior frontal gyrus (IFG)] compared to non-persuasive arguments [16]. 

On the other hand, similar brain regions are closely involved in moral reasoning, including DLPFC, orbitofrontal cortex (OFC), vmPFC, anterior cingulate cortex (ACC), precuneus, TPJ, parietal lobe, and superior medial prefrontal cortex [17,18,19]. Among these, the vmPFC plays a key role in regulating emotional responses tied to moral decisions and personal moral values. The orbitofrontal cortex contributes to evaluating morally relevant information, especially linked to guilt, while the TPJ and precuneus are primarily involved in understanding others’ perspectives and moral intentions.

However, most of the studies focusing on the neural correlates of both persuasion and moral reasoning used neuroimaging techniques, whereas, from a methodological level, electroencephalography (EEG) stands out for its excellent temporal resolution, affordability, and easy application, making it a widely accessible and precise tool for monitoring brain activity over time. By analyzing distinct EEG frequency bands (i.e., delta, theta, alpha, beta, and gamma) and considering their functional relevance, spatial distribution on the scalp, and lateralization across hemispheres, researchers can investigate neural patterns associated with specific cognitive and emotional mechanisms involved in persuasive interaction.

Notably, a study exploring EEG brain activity of dyads during a persuasive interaction aimed at promoting groupness [20] revealed a significant increase in neural dissimilarity between persuaders and receivers in the frontal regions, specifically within the delta and theta frequency bands. This effect was consistent across dyads, regardless of how effective the persuasion was perceived to be, suggesting that the persuasive processes rely on a cognitive and emotional asymmetry between interaction partners. The frontal dissimilarity highlights the persuader’s dominant and effortful role, characterized by increased attentional and emotional engagement, while the receiver maintains a more passive role.

To date, no research, to the best of our knowledge, has specifically investigated persuasive interactions related to moral decision-making through an EEG hyperscanning paradigm. The present study, therefore, aimed to examine how neural activity differs between two individuals—namely, the Persuasive Agent (PA) and the Persuasion Target (PT)—involved in a persuasive interaction by categorizing dyads based on the extent of perceived viewpoint change following the interaction.

To address this aim, participants took part in a naturalistic persuasion task (i.e., engaging in a spontaneous, face-to-face conversation without scripts or constrained responses), wherein they discussed a moral decision made by a fictional character in a realistic context. After reading the scenario, both the PA and the PT selected what they considered the most appropriate resolution to the moral dilemma from a set of predefined options. Subsequently, the PA attempted to persuade the PT that their chosen solution was the most suitable. EEG data were continuously recorded from both participants throughout the entire interaction.

Based on these premises, we anticipate that greater activation dissimilarity will emerge in the frontal regions compared to other cortical areas and specifically in low-frequency bands (theta and delta), reflecting affective processing, attentional engagement, and the integration of emotional and cognitive information, mechanisms central to both persuasive communication and moral decision-making [21,22,23,24]. Furthermore, we expect this pattern of frontal dissimilarity to be more pronounced in dyads where the perceived viewpoint change following the interaction was different between the two members of the dyad, reflecting greater divergence in the processing and internalization of persuasive messages.

## 2. Materials and Methods

### 2.1. Sample

The present study involved 14 homologous gender dyads of student volunteers (8 females: mean age = 23.5 years, SD age = 2.07 years; 6 males: mean age = 23.75 years, SD age = 2.01 years), who were recruited using a non-probability convenience sampling method. A priori power analysis, conducted using G*Power version 3.1.9.7, determined that a minimum of 27 participants was sufficient for repeated-measures ANOVA with six measurements and three groups (effect size f = 0.25, α = 0.05, power = 0.85, ε = 1). Therefore, the final sample size of N = 28 was deemed adequate for testing the study hypotheses. Participants, who were genetically unrelated and had no prior acquaintance before the experiment, were randomly assigned to one of the two experimental roles (PA or PT), resulting in 14 same-gender pairs. Exclusion criteria included a history of psychiatric or neurological disorders, significant depressive symptoms or impaired cognitive function, as well as short- or long-term memory deficits or taking psychoactive drugs. At the initial screening and recruitment phase, none of the 28 participants presented any of the above-mentioned exclusion criteria; therefore, they were all ultimately enrolled and satisfied the requirements for participation.

Homologous (same-gender) dyads were intentionally selected to reduce variability associated with gender differences in communication style, emotional expressiveness, and neural coupling, as widely documented in hyperscanning and interpersonal synchrony research [25,26,27,28]. Furthermore, because interpersonal synchrony is strongly modulated by preexisting relational closeness—strangers, friends, and romantic partners show distinct synchrony patterns across emotional contexts [29]—only genetically unrelated participants with no prior acquaintance were paired, to minimize relational variability and ensure greater homogeneity in dyadic interaction.

The present study was conducted in accordance with the ethical principles outlined in the Declaration of Helsinki (2013) and followed the General Data Protection Regulation (GDPR, EU Reg. 2016/679). Ethical approval was provided by the Ethics Committee of the Department of Psychology, Catholic University of the Sacred Heart, Milan, Italy.

### 2.2. Experimental Procedure

**Figure 1:**
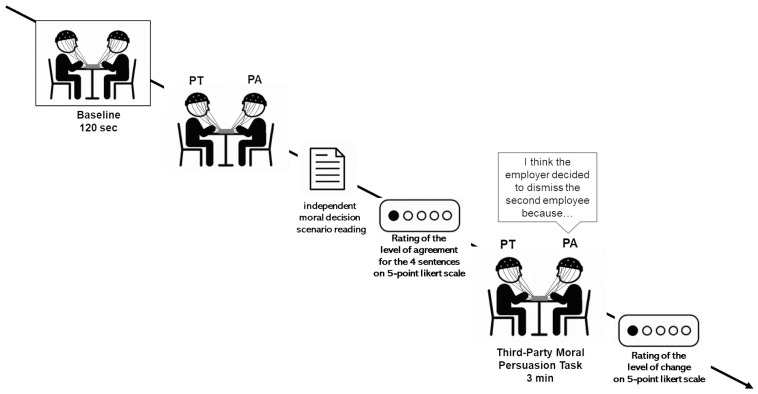
Experimental procedure. Graphical representation of the entire procedure, with baseline and the Third-Party Moral Persuasion Task.

Participants were seated close to each other to facilitate face-to-face interaction and minimize external interference. Before the beginning of the experiment, all participants gave their informed consent and were provided with comprehensive instructions to ensure clarity and compliance with the study guidelines. Moreover, participants were instructed to maintain calm, clearly articulate their speech (avoiding whispering), and take turns to ensure that the dialogue did not overlap. To prevent any influence of non-verbal cues on the outcome, video recordings were also used to monitor any significant changes in facial expressions or body posture. The neural activity of the dyads was recorded using an EEG hyperscanning paradigm, collecting data during both the 120-s baseline and the interaction (Figure 1).

#### Third-Party Moral Persuasion Task

In the Third-Party Moral Persuasion Task (TPMPT), participants were engaged in a persuasive communication, in which they were randomly assigned to the role of PA or PT. Specifically, participants were asked to evaluate a third-party decision. The task was introduced to the dyad by the following scenario: “The owner of a company must decide which of two employees to fire, each with a different profile and history. You are asked to evaluate and interpret the boss’s decision and the reasons behind it.”

First of all, both members of the dyad were invited to read the moral decision scenario independently. Then, they were asked to rate their level of agreement with a set of statements reflecting potential moral motivations behind the decision of the boss (e.g., “The employer decided to fire the second employee because he did not want to bear the responsibility of putting a family man in serious financial difficulty”; “The employer chose not to fire the first employee, as he has been with the company for many years and remains a valuable resource due to the significant investment made in his training”) through a 5-point Likert scale. Following this, the PA was instructed to select the statement that, in his/her view, best represented the true motivation of the boss and then was asked to explain to the PT his/her choice using a fixed formulation that started with “I think the employer decided to dismiss the second employee because…” In this step, the PA had to persuade the other member within 3 min that his/her motivation was the most accurate and morally justified explanation of the boss’s behavior. After this, both participants had to rate their level of agreement again, using the same set of predefined statements presented before the interaction.

To measure whether participants perceived a change in their initial attitude after the discussion, they were asked to complete a 5-point Likert scale, reflecting the degree to which they felt their viewpoint had changed as a result of the persuasive exchange, ranging from 1 (“strong disagreement”) to 5 (“strong agreement”). This allowed us to investigate whether and to what extent the interaction between the subjects facilitated a process of reconsideration or reorientation of the initial position and allowed us to define the variable “perceived viewpoint change” (PVC) among the dyads. The mean response of the sample was calculated from the responses of each member, and the dyads were categorized based on the perceived viewpoint change between PA and PT in three levels: high perceived viewpoint change (both members have a high perception of having changed their opinion; N = 5); mixed perceived viewpoint change (one member has a high perception and the other a low perception of having changed their opinion; N = 4); and low viewpoint change (both members have a low perception of having changed their opinion; N = 5).

### 2.3. EEG Data: Acquisition and Processing

EEG data were recorded during both the Baseline (BL) and TPMPT, using NEUROSCAN 4.2 software and a 16-channel DC amplifier (SYNAMPS system). Fifteen Ag/AgCl electrodes were placed according to the international 10/20 system [30] using an ElectroCap, with recordings referenced to the earlobes. Electrode sites included Fp1, Fp2, F3, F4, Fz, Cz, C3, C4, T7, T8, Pz, P3, P4, O1, and O2. In addition, two EOG electrodes were placed on the outer left eye to monitor eye movements. The electrode impedance was kept below 5 kΩ. Data were sampled at 1000 Hz and filtered with a 50 Hz notch filter.

EEG pre-processing was performed using Brain Vision Analyzer 2.0 (Brain Products GmbH, Gilching, Germany). A 0.01–50 Hz bandpass IIR filter (48 dB/octave) was applied, and the continuous signal was segmented into two-second epochs. These segments were visually inspected to exclude those containing ocular, muscular or motion artifacts.

In the clean segments, the power spectral density (PSD) was calculated for both conditions (BL and TPMPT) using a fast Fourier transform (FFT) with a Hamming window and a resolution of 0.5 Hz. The PSD was calculated for five standard EEG frequency bands: Delta (0.5–3.5 Hz), Theta (4–7.5 Hz), Alpha (8–12.5 Hz), Beta (13–30 Hz) and Gamma (30.5–50 Hz).

**Figure 2:**
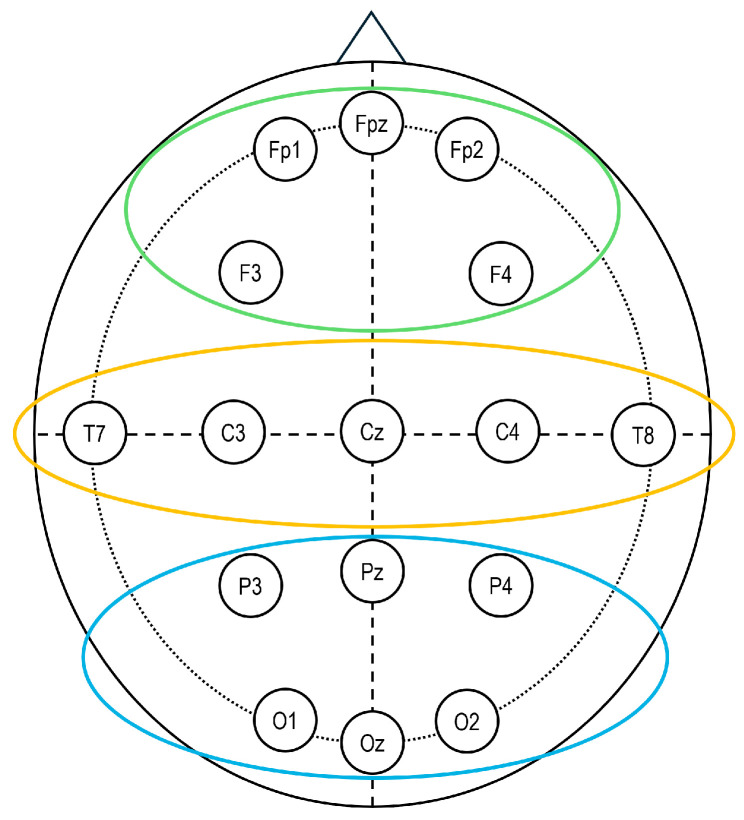
Electroencephalography (EEG). Sixteen-channel EEG montage adopted in the study, according to the 10/20 system of electrode placement and the selected regions of interest: frontal (Fp1, Fp2, AF3, AF4; in green), temporo-central (C3, C4, T7, T8; in yellow), and parieto-occipital (P3, P4, O1, O2; in blue).

Finally, for statistical analysis, the persuasion interaction was weighted over BL values [(PSD_TPMPT_ − PSD_BL_)/PSD_BL_]. Three Regions of Interest (ROIs) were defined to encompass the frontal, temporo-central, and parieto-occipital regions, which were computed by averaging data from the following electrodes: frontal (Fp1, Fp2, F3, F4), temporo-central (C3, C4, T7, T8), and parieto-occipital (P3, P4, O1, O2) (Figure 2).

### 2.4. Data Analysis

The current study examined dyadic dynamics by assuming that the PA and PT during interactions could be differentiated based on distinct neurophysiological activation patterns observed during the interaction and based on the perceived viewpoint change. To assess dissimilarity in PSD across frequency bands (Delta, Theta, Alpha, Beta, Gamma), Ward’s method of Euclidean distance (Eudis) between Region of Interest (ROI) (frontal, temporo-central and parieto-occipital) and Lateralization (left and right) for EEG bands within each dyad was used, serving as a dissimilarity index.

Before conducting statistical analyses, skewness and kurtosis tests were conducted to verify the data’s normality. Then, five sets of repeated-measures ANOVAs were conducted separately for the Eudis of each EEG frequency band (delta, theta, alpha, beta, and gamma) as a distinct dependent variable. ROI (3: frontal, temporo-central, parieto-occipital) and Lateralization (2: left, right) were included as within-subject factors and PVC (3: low, mixed, high) as a between-subjects factor.

Where necessary, degrees of freedom in ANOVA tests were corrected for sphericity violations using the Greenhouse-Geisser epsilon. Pairwise comparisons were used to test for significant interactions. Bonferroni correction was applied to control for the risk of type I error due to multiple comparisons. Effect sizes for significant results were reported using partial eta squared (η^2^p), and statistical significance was determined using an alpha level of 0.05.

## 3. Results

From a behavioral perspective, the results showed that the persuasive interaction was effective: in 10 out of 14 dyads, at least one participant changed their viewpoint following the discussion. This behavioral effect was further explored using the PVC measure, resulting in 5 dyads with high perceived change, 4 dyads with mixed perceived change, and 5 dyads with low perceived change.

### Delta

For the delta band, results showed a significant main effect for ROI (F[2,22] = 14.242; *p* < 001; η^2^p = 0.564), with higher dissimilarity for Delta in the frontal area compared to the temporo-central area (*p* = 0.002) and compared to the parieto-occipital area (*p* =0.007).

**Figure 3:**
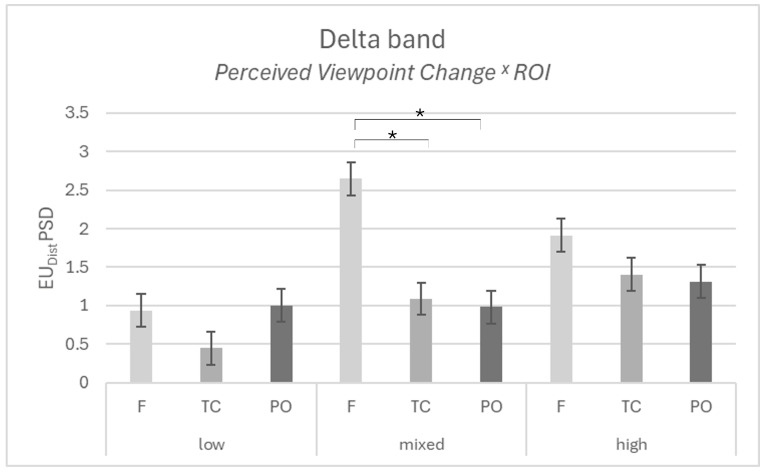
EEG results. The graph represents the significant interaction effect in Perceived Viewpoint Change x ROI for the Delta band in the mixed dyads. Bars represent ±1 standard error, and the star (*) marks a statistically significant comparison between the frontal area and the temporo-central and parieto-occipital areas.

Furthermore, an interaction effect was found for PVC × ROI (F[4,22] = 4.743; *p* = 0.007; η^2^p = 0.463), with higher dissimilarity for Delta in the frontal area compared to the temporo-central area (*p* = 0.012) and compared to the parieto-occipital area (*p* = 0.008), for the mixed dyads (Figure 3). No other significant effects were found for delta (all *p* > 0.05).

No significant effects were found for the Theta, Alpha, Beta, and Gamma bands (all *p* > 0.05).

## 4. Discussion

This study examined dissimilarity in neurophysiological activity within dyads, in which one member assumes the role of the PA and the other of the PT while engaged in a persuasive interaction involving a moral decision-making scenario and later categorized based on whether they reported a change in their initial decision.

EEG results showed an increased dissimilarity in delta activity over frontal areas compared to both the temporo-central and the parieto-occipital areas. Specifically, such increased dissimilarity over frontal area was higher in the mixed dyads (i.e., one member has a high perception and the other a low perception of having changed their opinion).

Delta oscillations play a crucial role in mechanisms of emotional regulation. Notably, variations in delta band activity are linked to the processing of both positive and negative interpersonal experiences, supporting the idea that delta frequencies contribute to heightened attentional engagement with emotionally salient stimuli [31]. Similarly, changes in delta activity may signal internal emotional states and their regulation [32]. These findings are consistent with evidence showing enhanced delta activity during tasks that demand emotional processing. Furthermore, prior evidence has shown that frontal delta band activation is linked to emotional processing, motivation, and response planning in challenging situations, all of which are critical during communicative decision-making [33,34].

Therefore, building on this evidence, we might speculate that the increased dissimilarity observed over frontal areas in mixed dyads could reflect variations in emotional and motivational responses between the two members. Indeed, given that the perceived viewpoint following the interaction was different between the members of the dyad, this increased neural dissimilarity might signal a greater divergence in the processing and internalization not only of the persuasive message but also of the moral decision-making process. This contrasts with the high and low dyads, in which both members either changed or maintained their perceived viewpoints in a more similar manner.

Therefore, our result, showing increased frontal delta band dissimilarity in mixed dyads (where only one member changes their opinion), can be interpreted as indicating greater divergence in emotional and motivational responses between partners. Rather than reflecting the internal state of a specific individual, this pattern suggests that the two members of mixed dyads differ more strongly in how they process the persuasive message and the moral content of the interaction. Neural dissimilarity is a relational measure, and in line with hyperscanning research, inter-brain divergence is typically understood as reflecting differences in partners’ engagement or processing modes rather than individual psychological states [3,13,31]. Evidence from persuasive and asymmetrical communicative interactions further shows that such divergence is a characteristic feature of these dynamics [16,20]. Specifically, the frontal delta activation likely reflects differentiated emotional and attentional engagement: the member changing his/her viewpoint may be more emotionally and motivationally invested in processing the persuasive message and the moral decisional statements, while the other may remain more passive or resistant. This aligns with previous studies suggesting a role asymmetry in persuasive interactions [16,20]. Indeed, the increased dissimilarity in mixed dyads, as compared to high- and low-change dyads (where both members either change or do not), suggests that higher emotional and motivational synchrony leads to more aligned opinions, while emotional and motivational dissonance results in divergent outcomes. Thus, our findings extend previous work by suggesting that frontal delta dissimilarity can index divergent emotional and motivational engagement in asymmetric persuasive interactions, as highlighted by recent hyperscanning studies.

It is also interesting to consider the lack of significant dissimilarity observed in the temporo-central and parieto-occipital regions in members of the dyad. Notably, such a pattern of activation can be interpreted as a reflection of their similar involvement in perspective-taking processes, which are central to moral decision-making. These areas, including the TPJ and the precuneus, are crucial for understanding others’ perspectives, moral intentions, and self-referential thinking [15,18]. In moral decision-making, both participants may be engaged in similar processes of considering the moral implications of the persuasive message or evaluating the perspectives of others in the interaction, thus likely activating these regions in a similar way as they engage in perspective-taking.

## 5. Conclusions

Overall, this study explored dissimilarity in neurophysiological activity within dyads during a persuasive interaction involving a moral decision-making scenario, categorizing them based on whether they reported a change in their initial decision. EEG results revealed increased delta activity dissimilarity in frontal areas, particularly in mixed dyads, where one member perceived a great change in opinion while the other perceived a low change. The observed increase in frontal delta dissimilarity may reflect divergent emotional and motivational states within the dyad, with the participant who changes viewpoint showing greater engagement while the other remains more passive, consistent with previous findings of asymmetry in persuasive interactions [16,20]. In contrast, the absence of dissimilarity in temporo-central and parieto-occipital regions, critical for understanding others’ perspectives, suggests similar neural activation, likely related to joint engagement in perspective-taking processes essential for moral reasoning [18,35].

Despite the study’s contribution to the literature on persuasion in moral decision-making, there are several limitations that must be considered. Firstly, future studies should include a more diverse range of participants, with varying characteristics or experiences, which could impact persuasive interactions. Secondly, to gain deeper insights into the factors influencing persuasive interactions and their effectiveness, future research could explore additional variables, such as personality traits. Finally, as shown in previous research on emotions and prosocial behavior [36], a more comprehensive understanding of the neurophysiological processes involved in persuasion could be achieved by adopting a multi-level approach, integrating EEG frequency band data, autonomic indices, and hemodynamic brain signals from functional Near-Infrared Spectroscopy (fNIRS) within an interacting dyad.

## Data Availability

The data presented in this study are available on request from the corresponding author due to ethical reasons for sensitive personal data protection (requests will be evaluated according to the GDPR—Reg. UE 2016/679 and its ethical guidelines).

## Abbreviations

The following abbreviations are used in this manuscript:

PA	Persuasive Agent
PT	Persuasion Target
TPMPT	Third-Party Moral Persuasion Task
PVC	perceived viewpoint change
